# Systematic Review of Sleep Characteristics in Myalgic Encephalomyelitis/Chronic Fatigue Syndrome

**DOI:** 10.3390/healthcare9050568

**Published:** 2021-05-11

**Authors:** Rebekah Maksoud, Natalie Eaton-Fitch, Michael Matula, Hélène Cabanas, Donald Staines, Sonya Marshall-Gradisnik

**Affiliations:** 1 National Centre for Neuroimmunology and Emerging Diseases (NCNED), Menzies Health Institute Queensland, Griffith University, Gold Coast 4222, Australia; natalie.eaton-fitch@griffithuni.edu.au (N.E.-F.); m.matula@griffith.edu.au (M.M.); h.cabanas@griffith.edu.au (H.C.); d.staines@griffith.edu.au (D.S.); s.marshall-gradisnik@griffith.edu.au (S.M.-G.); 2 Consortium Health International for Myalgic Encephalomyelitis, Griffith University, Gold Coast 4222, Australia; 3 School of Medical Science, Griffith University, Gold Coast 4222, Australia

**Keywords:** Myalgic Encephalomyelitis, chronic fatigue syndrome, sleep, polysomnography, multiple sleep latency testing

## Abstract

(1) Background—Myalgic Encephalomyelitis/Chronic Fatigue Syndrome (ME/CFS) is a multifaceted illness characterized by profound and persistent fatigue unrelieved by rest along with a range of other debilitating symptoms. Experiences of unrefreshing and disturbed sleep are frequently described by ME/CFS patients. This is the first systematic review assessing sleep characteristics in ME/CFS. The aim of this review is to determine whether there are clinical characteristics of sleep in ME/CFS patients compared to healthy controls using objective measures such as polysomnography and multiple sleep latency testing. (2) Methods—the following databases—Pubmed, Embase, Medline (EBSCO host) and Web of Science, were systematically searched for journal articles published between January 1994 to 19 February 2021. Articles that referred to polysomnography or multiple sleep latency testing and ME/CFS patients were selected, and further refined through use of specific inclusion and exclusion criteria. Quality and bias were measured using the Joanna Briggs Institute checklist. (3) Results—twenty observational studies were included in this review. The studies investigated objective measures of sleep quality in ME/CFS. Subjective measures including perceived sleep quality and other quality of life factors were also described. (4) Conclusions—Many of the parameters measured including slow- wave sleep, apnea- hypopnea index, spectral activity and multiple sleep latency testing were inconsistent across the studies. The available research on sleep quality in ME/CFS was also limited by recruitment decisions, confounding factors, small sample sizes and non-replicated findings. Future well-designed studies are required to understand sleep quality in ME/CFS patients.

## 1. Introduction

Myalgic Encephalomyelitis/ Chronic Fatigue Syndrome (ME/CFS) is a medical condition characterised by non-restorative, incapacitating fatigue that is unrelieved by rest in combination with a plethora of other symptoms such as neurological, immune and endocrine disruption [1]. Unrefreshing or disturbed sleep is an almost universal symptom reported in about 91% of patients in the absence of a primary sleep disorder (PSD) [1,2]. The presentation and severity of these symptoms ranges between patients and results in considerable loss of quality of life [3]. There currently remains no diagnostic test nor targeted treatment for this condition. Diagnosis is instead dependent on the application of symptom- specific case criteria following the exclusion of any other potential medical cause [4]. 

There are three main criteria used in research and clinical practice to diagnose ME/CFS and include: (1) The Center for Disease Control’s (CDC) Fukuda criteria (FC) (1994); (2) The Canadian Consensus Criteria (CCC) (2003) and (3), The International Consensus Criteria (ICC) (2011) [1,5,6]. Diagnosis with the FC is dependent on the presence of persistent fatigue that is unrelieved by rest in combination with four out of a potential eight additional symptoms including but not limited to unrefreshing sleep [5]. The revised CCC criteria builds upon the FC with emphasis on post-exertional malaise as a key symptom of ME/CFS. In this criteria, sleep disturbance was also described as a potential symptom of this disorder [6]. The ICC criteria divides sleep specific symptoms into two categories: disturbed sleep patterns and unrefreshing sleep and includes the most homogenous subset of patients [1]. The most recent institute of medicine criteria (IOMC) has unrefreshing sleep listed as one of the three required symptoms [4]. Unrefreshing or disturbed sleep can include the following sleep characteristics: reversed sleep rhythms and frequent awakenings [7]. 

Polysomnography (PSG) is the most common objective measure of sleep quality. PSG measures at various sleep phases including rapid eye movement (REM) and non-REM sleep. Non-REM sleep phases include: stage N1, N2 and N3/4 or Slow wave sleep (SWS). SWS is the deepest phase of non-REM sleep [8]. Other parameters including sleep onset latency (SOL) which is the time taken from being fully awake to fast asleep as well as apnoea- hypopnoea index (AHI) and microarousal Index (MAI) values [9]. AHI values are the number of apnoea and hypopnea events per hour of sleep. MAI values allows measurement of sleep fragmentation [10]. Multiple Sleep Latency Testing (MSLT) an objective measure to assess the ability to fall asleep under controlled conditions is at times used in combination with PSG [11]. 

This is the first systematic review to critically appraise primary studies that assess objective measures of sleep quality in ME/CFS patients using PSG and/or MSLT. Secondary to this, subjective measures including sleep quality and depression scores were also evaluated. 

## 2. Methods

This study was conducted according to Cochrane reviews and Preferred Reporting Items for Systematic Reviews and Meta-analyses 2020 (PRISMA 2020) guidelines [12,13]. To ensure that international standards were maintained when reporting information in this systematic review these guidelines were used. Four electronic databases (Pubmed, Medline [EBSCOHost], Embase and Web of Science) were systematically searched. Articles containing the following medical Subject Headings (MeSH) terms “Syndrome, Chronic Fatigue” [Mesh] AND (‘Multiple Sleep Latency Test*’ OR ‘Polysomnography’ OR ‘Polysomnograph*’) were searched between January 1995 and 19th February 2021 (full list of terms can be found in Table S1). Terms were combined with the Boolean operators ‘AND’ in order to tie the disease of interest with objective measures of sleep quality and ‘OR’ to expand the search for all expressions of cases. Two identical literature searches were conducted separately by two different authors. Citation searching was completed, and no additional papers were selected. Searching for unpublished literature was not performed. No additional papers were identified in the final search or through alternative databases such as Griffith University institute library or Google Scholar. 

### 2.1. Inclusion/ Exclusion Criteria 

Studies were included in the review if they contained two or more of the key search terms in the abstract or title and adhered to the following inclusion criteria: (i) published in 1995 or later as the FC was established in December 1994; (ii) human participants who were aged 18 years or over; (iii) full- text articles written in English; (iv) were observational studies reporting on original research; (v) ME/CFS was defined according to the following case criteria: FC (1994), CCC (2003) or ICC (2011) and IOMC (2015); (vi) all studies investigated objective measures of sleep quality. 

Articles were excluded from this review if they did not include at least two key search terms in the abstract or title or if they had any of the following exclusion criteria: (i) written prior to the introduction of the FC in 1994; (ii) conducted in participants that were under 18; (iii) articles not written in English or weren’t available as full-text; (iv) were interventional based or reported on non-original data including: duplicate studies, case reports or review articles; (v) use of alternative case criteria; (vi) studies were not relevant to the scope of this review. (vii) Publications were also excluded if the ME/CFS cohort was compared with another patient group (e.g., fibromyalgia, depression etc.) and not compared with HC. 

### 2.2. Selection of Studies

The referencing management software package Endnote X9 was used to screen, sort and store all articles from the databases. Duplicates were removed using Endnote’s automatic feature. The title and abstract of each article were screened for selected keywords and those which did not contain at least one ME/CFS keyword and one sleep test keyword. The remaining articles that also adhered to inclusion and exclusion criteria were selected. This process was independently conducted by RM and MM. There were minor differences between the two authors, however, these were discussed, and a final list was compiled and approved by both authors. The final list was then reviewed and deemed accordant by all other authors.

### 2.3. Data Extraction

The following data was extracted from the included studies: (1) diagnostic criteria; (2) study design; (3) sample size; (4) age; (5) sex; (6) BMI; (7) total sleep duration; (8) method of analysis; (9) primary outcomes; (10) secondary outcomes. 

### 2.4. Quality Analysis

All publications included in this systematic review were evaluated for quality and bias using the Joana Briggs Institute (JBI) Critical Appraisal Checklist for Case Control Studies (CACCCS) (File S1. JBI CACCCS and justification). This checklist was selected due to it being an internationally recognised and validated method of evaluating study quality and bias. Quality assessment was separately completed by two authors (RM and NEF). As Item four, five and nine were intervention based these items were excluded in all studies except one [14].

## 3. Results

**Figure 1:**
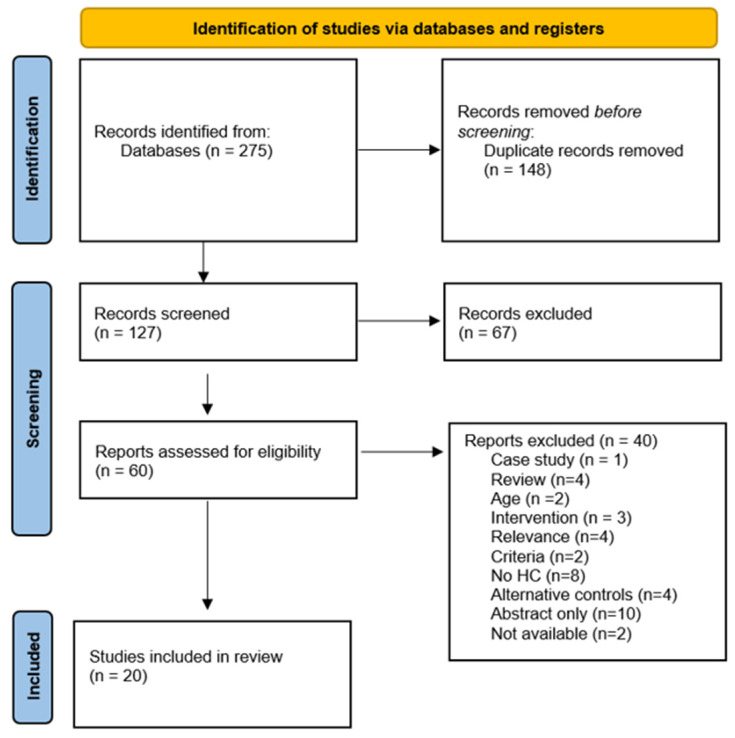
PRISMA 2020 flow diagram of literature search for included studies in this review of sleep and ME/CFS.

Using the selected search terms, a total of 275 papers were identified using the following databases: Embase (108), Pubmed (50), Medline (61), and Web of Science (56). Following the screening process the total number of papers was 20. A detailed outline of the search process is presented in Figure 1. All included papers investigated objective measures of sleep quality in ME/CFS patients compared to HC.

### 3.1. Participant and Study Characteristics 

Participant and study characteristics are presented in Table S2. Four (20%) out of the 20 articles included in this review were observational twin studies [15,16,17,18]. The remainder of the included articles (80%) were observational case-control studies [9,14,19,20,21,22,23,24,25,26,27,28,29,30,31,32]. Across all studies, the mean number of ME/CFS patients and HCs included was 26.6 and 24 respectively. Majority of the included participants were female (87%). The mean age across all studies was 41.5 for the ME/CFS group and 39.2 for the HC group [9,14,15,16,17,18,19,20,21,22,23,24,25,26,27,28,29,30,31,32]. Fifteen out of 20 papers reported a value for body mass index (BMI) [9,14,15,19,20,21,22,23,24,25,26,27,28,31,32]. The average BMI was 25.5 for ME/CFS patients and for 25.4 HC [9,14,15,19,20,21,22,23,24,25,26,27,28,31,32]. The FC was used in all studies to diagnose participants [9,14,15,16,17,18,19,20,21,22,23,24,25,26,27,28,29,30,31,32]. One study, however, used both the FC criteria and the CCC to diagnose [32]. Average total sleep time was 397.03 min for ME/CFS patients and 400.1 min for the HC group [9,14,15,16,17,18,19,20,21,22,23,24,25,26,27,28,29,30,31,32]. 

### 3.2. Literature Reporting Changes in Objective Sleep Measures

Objective sleep measures are presented in Table S3. Two twin studies identified an increase in REM sleep in ME/CFS patients compared to their healthy twin [16,17]. One article reported significantly reduced REM to non-REM sleep stage transitions [30]. Alteration of transition patterns resulting in greater relative transition frequency was also observed [30]. Sleep onset latency (SOL) was investigated in 13 articles [9,14,17,19,21,22,23,24,25,26,27,28,31]. All 13 papers reported no differences in SOL between the ME/CFS patients and the HC [9,14,17,19,21,22,23,24,25,26,27,28,31]. Non-REM sleep stages, (NREM) including stage 1- 4 sleep, % was investigated in 12 studies [9,14,15,16,17,19,21,22,25,26,29,31]. Two of the 12 studies reported increased stage 3 sleep, % [16,22]. All other findings were insignificant [9,14,15,17,19,21,25,26,29,31]. There were 11 studies that investigated slow-wave sleep (SWS) duration [9,14,15,21,22,23,24,25,28,29,31]. From these studies, only three found that SWS in ME/CFS was significantly longer in duration compared to HC [24,25,28]. The remaining studies reported no difference between the two groups [9,14,15,21,22,23,29,31]. 

When assessing sleep apnoea characteristics, five studies detected no differences in AHI [14,15,21,22,23]. Three studies detected differences in AHI [16,24,31]. MAI was measured in five studies [14,23,24,25,31]. An increase of MAI in ME/CFS patients was found in all the studies [14,23,24,25,31].

### 3.3. Literature Reporting Changes in Spectral Activity 

Three articles investigated spectral activity during sleep [15,20,22]. A twin study found no significant differences in spectral power in any frequency band assessed: REM latency, delta-wave, fast frequency beta or alpha power between the twin with ME/CFS and the healthy twin [15]. Another study showed that there was diminished alpha power during stage 2, slow wave, and REM sleep in the ME/CFS cohorts compared to HC [20]. Delta power was found to be decreased during SWS but then was elevated during stage 1 and REM in the ME/CFS cohort. Theta, sigma and beta spectral power during stage 2, SWS and REM were significantly reduced in patients compared to HC [20]. One article found that ultra-slow delta power was significantly lower in ME/CFS patients compared to HC during N3 sleep while all other frequencies tested: theta, alpha, sigma and beta did not differ [22].

### 3.4. Literature Reporting Changes in MSLT 

Changes in MSLT were investigated in six articles [18,19,20,23,24,26]. One study found reduced mean sleep latency on MSLT in ME/CFS patients compared with HC [23]. Another study found a negative correlation between individual Epworth Sleepiness Scale (ESS) and mean latency scores in both groups [18]. All other articles investigating MSLT identified no significant differences between ME/CFS patients and HC [14,19,26]. 

### 3.5. Literature Reporting Changes in Secondary Outcomes

Participant and study characteristics are presented in Table S4. Various secondary outcome measures were investigated in 14 out of 20 included studies [9,14,17,18,19,22,23,24,25,27,28,29,31,32]. Additionally, different tools were used to measure the same outcomes. Subjective sleep quality or sleepiness was measured in 13 of the studies [9,14,17,18,19,22,23,24,25,28,29,31,32]. All these studies reported significant differences in sleep quality or perceived sleepiness in ME/CFS patients compared with HC. Depression scores were significantly higher in all six studies that included values [14,24,25,27,31,32]. In the five studies that measured anxiety, the ME/CFS scores were significantly different from HC in all but one study [14,23,24,31,32]. Insomnia was investigated in two studies and was found to be significantly higher in ME/CFS patients compared with HC [18,19]. Fatigue levels were also significantly greater in ME/CFS patients in all seven studies that measured this variable [14,21,23,24,25,28,31]. One study investigated emotional awareness in ME/CFS patients compared with HC [32]. Significant differences in some emotional awareness parameters including TAS-20, TAS total and LEAS-self were found and these correlated with number of awakenings in ME/CFS patients [32]. 

### 3.6. Quality Assessment 

The Joanna Briggs Institute (JBI) Critical Appraisal Checklist for Case Control Studies (CACCCS) was used to review the selected articles quality and bias. Justification can be found in file S1. Item 4, 5 and 9 were excluded in all studies except Neu 2014B [14]. The study included an exposure to a cognitive test. The authors successfully measured the effect of the exposure for an appropriate duration in a standard, valid, and reliable way across patients and HC [14]. Item 8 was most frequently addressed where 100% of the studies assessed outcomes in a standard, valid and reliable way [9,14,15,16,17,18,19,20,21,22,23,24,25,26,27,28,29,30,31,32]. Nineteen out of 20 studies successfully identified confounding factors [9,14,15,16,17,18,19,20,21,22,23,24,25,26,27,28,29,30,31]. The confounding factors that were addressed were effectively mitigated in 17 of the studies [14,15,16,17,18,19,20,21,22,23,24,25,26,28,29,30,31]. Sixteen studies had appropriately matched patients and HC [14,15,16,17,18,19,20,22,23,24,26,27,28,29,31,32]. Nineteen articles utilised consistent criteria to identify ME/CFS patients and HC [9,14,15,16,17,18,19,20,21,22,23,25,26,27,28,29,30,31,32]. Item 2 was the least addressed item where only seven studies appropriately defined and matched source population for ME/CFS patients and HC [9,15,16,17,18,19,20]. Thirteen of the articles included appropriate statistical analysis [9,14,19,20,21,22,24,25,27,28,30,31,32].

## 4. Discussion

ME/CFS patients report a significant number of sleep complaints [9,14,15,16,17,18,19,20,21,22,23,24,25,26,27,28,29,30,31,32]. The aim of this systematic review was to investigate primary studies that assess objective measures of sleep quality in ME/CFS patients using PSG and/or MSLT compared with HC. Subjective scores including depression, anxiety and QOL scores were also measured. Variable results from these studies were found. 

This is the first systematic review assessing objective measures of sleep quality in ME/CFS patients with respect to HC. This method allows the inclusion of all relevant articles. A review of sleep in ME/CFS patients however was undertaken by Jackson et al. [7]. The major findings reported in this publication include: objective and subjective contrasts in sleep quality as well as early evidence suggesting differences in sleep stage transitions, sleep instability and heart rate variability in ME/CFS patients compared with HC [7]. This review was published in 2012, therefore, a significant amount of time has passed since its publication [7]. Additional studies, in comparison to Jackson et al. have also been identified through this systematic review process [7,9,14,17,22,23,24,25,27,32]. A subset of sleep studies was also included in review in a neuroimaging paper by Maksoud et al. [33]. This current systematic review is important as it brings a complete and up-to-date picture of sleep and ME/CFS. 

The average age of patients in the included studies of this systematic review was 41.5 years. Approximately 87% of the patients were female. This is consistent with literature showing that ME/CFS is most frequently reported in females aged between 29–35 years [34,35]. This current systematic review selected for participants over the age of 18 due to age-related differences in sleep [15,27,30]. The included studies had a maximum age cut-off for the same reason. Some studies (15%) only recruited females to account for sex- specific differences in sleep as well as to reduce patient pool heterogeneity [15,27,30]. Six of the studies included information on race or ethnicity where majority of the participants were Caucasian [16,17,18,19,20,26]. There was no significant difference in total sleep time between ME/CFS patients and HC. Selected studies restricted outliers of total sleep time in either group to control for potential sleep-related morbidities. 

Four of the included studies were twin-based [15,16,17,18]. Recruitment of twins assists in moderating differences in genetic and environmental factors. The genetic contribution and potential familial vulnerability of ME/CFS on the unaffected twin is not currently known [15,16,17,18]. Ball et al. reported sleep disruption in both ME/CFS patients and their unaffected twin [16]. Therefore, future considerations may involve comparative studies with closely-matched non-relative controls to ensure that there is no genetic contribution to sleep disruption in the selected HC [16]. 

Paediatric and adolescent sleep characteristics have not been captured in this sleep review due to potentially significant age-related differences. Presentation of illness may also differ between adults and children [36]. Case criteria have also described unrefreshing sleep as a hallmark symptom [1,4]. One study was identified during the screening process that investigates sleep in adolescent ME/CFS patients [37]. This study found that there were significantly higher levels of sleep disruption in adolescents with ME/CFS, and includes brief and longer awakenings [37]. Further investigation of sleep disruption in paediatric and adolescent ME/CFS populations is required. 

All of the included studies utilised the FC to classify ME/CFS patients [9,14,15,16,17,18,19,20,21,22,23,24,25,26,27,28,29,30,31,32]. One study used both FC and CCC [32]. Compared to the later definitions, the FC is considered too broad and often presents with a heterogenous subset of patients [4]. Consideration of future studies may include representation of patients diagnosed with more stringent definitions [1,4,6]. The more recent case definitions incorporate ME/CFS specific symptoms such as post-exertional malaise that allows a more representative subset of ME/CFS patients to be included [1,4,6]. 

A limitation to this systematic review is that it was restricted to articles that had PSG and MSLT in the abstract or title [9,14,15,16,17,18,19,20,21,22,23,24,25,26,27,28,29,30,31,32]. These terms were selected on the basis of being the primary objective measure of sleep used. Other measures that may describe sleep quality include actigraphy, observation, bed sensors, eyelid movement- and non-invasive arm sensors [38]. Reports on the use of actigraphy for measures investigated in this paper including sleep-wake cycles are controversial. These terms were also excluded due to their broad nature, although this may have resulted in potentially relevant articles not being captured. Some studies also utilised components of polysomnography including EEG and discussed features of sleep but did not undergo the whole polysomnography process [39]. Additionally, two studies by Neu et al. were not included in this review due to not containing any key words in the abstract or title [40,41]. These papers followed most of our selection criteria. One used PSG to assess cognitive impairment in ME/CFS [40]. ME/CFS performance in almost all cognitive tasks was lower compared with HC. EEG theta power was also significantly higher in ME/CFS patients. The other paper investigated sleep parameters in ME/CFS compared with HC and primary sleep disorders [41]. ME/CFS showed higher slow-wave sleep, however this is an inconsistent parameter across studies included in this review. In order to avoid selection bias this paper could not be handpicked to include in our study based on recommendations of Cochrane guidelines handbook [12,40,41]. 

Existing comorbid disorders may also play a role on sleep disruption in ME/CFS patients. Fibromyalgia syndrome (FMS), migraine and irritable bowel syndrome (IBS) all commonly occur in ME/CFS patients and have known implications on sleep efficiency. PSG studies of FMS patients reported poorer sleep quality as well as higher number of awakenings, higher arousal index, greater AHI and lower N1 sleep in FMS patients compared to HC. Sleep disturbance also exacerbates symptom severity in FMS [42,43]. One included study separated patients with ME/CFS alone or comorbid ME/CFS and FMS. There was a higher number of cases of sleep disorders among those diagnosed with IBS, further analysis is required, however, to understand this relationship [44]. All of the studies did not include patients who had a Diagnostic and Statistical Manual of Mental Disorders, 4th Edition (DSM-IV) disorder. Therefore, the sleep patterns that are observed cannot be attributed to major depression episodes or other associated conditions [9,14,15,16,17,18,19,20,21,22,23,24,25,26,27,28,29,30,31,32].

Care needs to be considered to ensure that all sleep characteristics are related to ME/CFS specifically, not other associated disorders. Some studies recruited ME/CFS patients without comorbidities to confirm the results observed were representative of ME/CFS [21]. In these studies, a minority of ME/CFS patients exhibited abnormalities in PSG data. Some studies even further classified ME/CFS patients in of groups into less sleepy and sleepier groups; this was conducted in two of the studies [27,28]. 

Confounding factors including consumption of alcohol and caffeine, medication, strenuous exercise, or a change in time zones may have contributed to varied results observed. Nine of the studies accounted for alcohol and/or caffeine [14,15,16,17,18,25,27,28,30]. Three of the studies also ensured that participants were not travelling from conflicting time zones within a certain timeframe of the study or adjusted the sleep schedule according to their place of residence [9,15,22]. In three of the studies participants, in particular HC were requested to refrain from strenuous exercise in the daytime prior to being assessed at night [27,28,30]. Nine of the studies controlled for medication [9,15,16,17,18,19,20,22,26]. These confounding factors may have influenced changes in sleep scheduling or temporarily impair the participants ability to sleep. Therefore, to ensure consistency across the studies, controlling for these confounding factors is a necessary consideration for future studies. 

In the study conducted by Bileviciute-Ljungar et al. HC were included to measure emotional awareness parameters, however, they used previously recorded HC data for PSG comparisons and only conducted PSG recordings on patients [32]. It is important to include well-defined and matched controls for each study to ensure that there is consistency between groups and that all other experimental variables are appropriately controlled for [32,45]. 

Eleven out of 20 studies accounted for first night effects. Considerations included recording over consecutive days [9,14,15,16,17,18,20,21,22,23,26]. In a study examining the impact of first night effects in four groups of participants: sleep-related breathing disorders, insomnia, movement and behavioural disorders and HC, it was found that in all groups there was a significant first night effect [46]. Additionally, Le Bon et al. also investigated first night effects in ME/CFS patients and found clinically significant differences in PSG recordings including SPT, TST, Sleep Efficiency and REM Sleep that can be attributed to first night effects [47]. Recommendations from these studies included measuring participants sleep parameters for at least two consecutive nights to ensure that first night sleep effects are accounted for [46,47]. This is an important consideration for all sleep physiology studies. 

Two of the studies used a take-home PSG kit [9,29]. Using this method means that conditions are not controlled for including light exposure and sleep disruptions that may come from an uncontrolled setting. Use of take-home polysomnography kits allows participation of a greater proportion of ME/CFS patients that are housebound, bedbound, or otherwise unable to attend a research site. As approximately 25% of ME/CFS patients have more severe symptoms this is an important consideration [3]. Eighteen studies required participants to attend a sleep clinic [14,15,16,17,18,19,20,21,22,23,24,25,26,27,28,30,31,32]. Those who spent overnight in a sleep clinic will have more appropriately monitored process, however, change in sleep setting may also affect results. 

Investigations into other factors influencing sleep quality, including melatonin and other hormone levels, do not fall within the scope of this review as no interventional studies were analysed. Melatonin levels influence multiple physiological processes including immune cell pathways [48]. As the most consistent immunological feature of ME/CFS is reduced natural killer (NK) cell cytotoxicity, this area will benefit from additional research [49]. Dysregulation of 2-5A synthetase/RNase L antiviral pathway has been previously linked with sleep disruption in particular changes to alpha delta sleep, however, investigations by Van Hoof et al. did not support associations [50]. Van Hoof et al. was not included in our analysis because that study did not have a HC group [50]. Changes in other hormone profiles including the hypothalamic-pituitary axis (HPA) has also been implicated in ME/CFS pathogenesis. Dysregulation of HPA also has known implications on sleep [7]. 

As mentioned previously, intervention studies were not included in the scope of this review. Majority of the intervention studies that were captured by the search terms focused on implementing exercise or alternative sleep scheduling such as a four-hour sleep delay on ME/CFS patients [51,52]. Introducing these interventions at even a moderate capacity in ME/CFS patients may result in the exacerbation of symptoms including post-exertional malaise (PEM). Therefore careful study design to ensure patient safety must be incorporated [53]. A review of currently available literature on these intervention studies is yet to be conducted. 

Variable results were found for sleep apnoea scores in ME/CFS patients compared with HC. Le Bon et al. suggested that the percentage of patients with obstructive sleep apnoea may be influenced by the cut-off selected [21]. Some ME/CFS patients with comorbid sleep disorders have found benefits using a continuous positive airway pressure (CPAP) machine. This includes cognitive and daytime sleepiness. This machine, however, does not remediate the underlying fatigue [7]. A study conducted by Libman et al. has suggested that sleep apnoea-hypopnea syndrome should not be an exclusion criterion for ME/CFS; it instead should be considered a potential comorbidity [54]. Including participants with comorbid primary sleep disorders, however, makes distinguishing sleep patterns in ME/CFS patients difficult [21]. 

One study although finding no significant changes in PSG recordings reported higher fractal scaling index α1, a measure of heart rate variability during nonrapid eye movement (non-REM) sleep (Stages 1, 2, and 3 sleep) in the a.m. sleepier ME/CFS group compared with HC [27]. This suggests contribution of RR interval dynamics, an electrocardiogram parameter or autonomic nervous system activity during non-REM sleep to disrupted sleep in ME/CFS patients [27]. Additional studies have shown the potential role of cardiovascular regulation in the pathomechanism of ME/CFS [27,55]. ME/CFS patients presented with increased heart rate, and reduced heart rate variability. Orthostatic intolerance also promoted increased symptom severity [27]. These changes may suggest that there is dysregulation of the autonomic nervous system in ME/CFS pathology. These findings also demonstrate the importance of addressing whether unrefreshing sleep is a consequence of another underlying pathology in ME/CFS patients. Due to this feature observed in ME/CFS patients, it may be an important future consideration to further stratify patients on the basis of having postural tachycardia syndrome (POTS) or any other form of orthostatic intolerance [56]. This may further assist in understanding their contribution to sleep quality in ME/CFS patients. 

A report made throughout the studies was an increase in slow wave sleep. Ball et al., made an association of this finding with immunological changes in ME/CFS patients [16]. It was suggested that this feature may be related to the release of cytokines [16]. However, there is insufficient evidence on the role of cytokines in ME/CFS pathomechanism [57]. Some studies also showed that there were no differences in SWS in ME/CFS patients or that there were only changes following sleep challenge [51,58]. These studies, however, did not follow inclusion criteria and were not selected for review. 

The 2012 study by Le Bon et al. found that there was decreased ultra-slow delta power in ME/CFS patients compared with HC [22]. This result emphasised the importance of looking beyond conventional EEG bands and to exercise caution when categorising sleep EEG into discrete stages alone as some trends may be overlooked [22,33]. 

MSLT results were inconsistent across the studies. One out of six studies that used MSLT reported significant disruptions in ME/CFS patients compared with HC [23]. It has been suggested that the presence of a comorbid sleep disorder in addition to ME/CFS may contribute to excessive daytime sleepiness [23]. 

A common trend in these sleep studies is that there is a discrepancy between subjective sleep measures and objective sleep measures. This misperception was further investigated by Shan et al., who identified that there were structural changes in the medial prefrontal cortex that correlates with unrefreshing sleep in ME/CFS patients [59]. Approximately 91% of ME/CFS patients exhibit symptoms of unrefreshing sleep [59]. This finding shows the importance of using alternative neuroimaging techniques available to address sleep quality impairment in ME/CFS [59]. Additionally, sleep disruption can also be explained by additional abnormalities that have been described including brainstem reticular activation system connectivity deficits [59,60]. A majority of the studies utilise well-established sleep scoring tools, however, validation of some of these tools in ME/CFS populations is required. Additionally, the use of these tools may be affected by self-report bias [61]. Further research on the discrepancy between subjective and objective measures of sleep quality is required.

### Quality Assessment 

There were variable quality levels across the studies. Standard measures for clinical evaluation were used across all studies as PSG as well as MSLT in selected studies were employed. All studies included information on ME/CFS selection criteria, however, in some studies HC selection criteria were not provided. Item one was successfully addressed if two or more forms of patient and HC matching is employed including age, sex and BMI/weight-matching. A greater proportion of studies identified confounding variables and provided methods to mitigate them. Item two which assesses whether socio-demographic characteristics between ME/CFS patients and HC were appropriately matched was the least addressed item. Recommendations for future studies include reporting and matching of patient socio-demographics. 

## 5. Conclusions

In the five studies that investigated MAI, all studies showed an increase in this parameter. SOL and NREM were not significantly different between ME/CFS patients throughout the studies. Slow- wave sleep, AHI, spectral activity, and MSLT were inconsistent across the studies. These results require validation in future well-designed studies. Numerous considerations for future experiments have been recommended including recruitment of participants with more stringent ME/CFS criteria and controlling for first night effects. Effective control of confounding variables of sleep quality including medications, change in time zones or strenuous exercise can also be implemented to improve overall study design. Replication of these studies in larger well-matched populations is also required. 

## Supplementary Materials

The following are available online at https://www.mdpi.com/2227-9032/9/5/568/s1, Table S1: Search code, Table S2: Study and Patient Characteristics, Table S3: Summary of Primary Outcome Measures, Table S4: Summary of Secondary Outcome Measures, File S1: JBI CACCCS and justification.
