# Supplementary material for: Systematic Review of Sleep Characteristics in Myalgic Encephalomyelitis/Chronic Fatigue Syndrome
Source: Healthcare (Basel). 2021 May 11;9(5):568. doi: 10.3390/healthcare9050568 (PMC8150292; doi:10.3390/healthcare9050568)
Supplement: Supplementary file 1 [file healthcare-09-00568-s001.zip › Supplementary Files/File S1. JBI CACCCS and justification.pdf]

File S1. JBI CACCCS and justification

	1	2	3	4	5	6	7	8	9	10
Armitage et al.2009	Yes	Yes	Yes	N/A	N/A	Yes	Yes	Yes	N/A	No
Ball et al. 2004.	Yes	Yes	Yes	N/A	N/A	Yes	Yes	Yes	N/A	No
Bileviciute-Ljungar *et al*. 2020	Yes	No	Yes	N/A	N/A	No	No	Yes	N/A	Yes
Decker et al. 2009	Yes	Yes	Yes	N/A	N/A	Yes	Yes	Yes	N/A	Yes
Gotts et al. 2016	No	Yes	Yes	N/A	N/A	Yes	No	Yes	N/A	Yes
Kishi et al. 2008.	U	No	Yes	N/A	N/A	Yes	Yes	Yes	N/A	Yes
Le Bon et al. 2008	No	No	Yes	N/A	N/A	Yes	Yes	Yes	N/A	Yes
Le Bon et al 2012	Yes	U	Yes	N/A	N/A	Yes	Yes	Yes	N/A	Yes
Majer et al. 2007	Yes	Yes	Yes	N/A	N/A	Yes	Yes	Yes	N/A	Yes
Neu et al. 2007	Yes	No	Yes	N/A	N/A	Yes	Yes	Yes	N/A	Yes
Neu et al. 2008	Yes	No	Yes	N/A	N/A	Yes	Yes	Yes	N/A	No
Neu et al. 2014A	Yes	No	No	N/A	N/A	Yes	Yes	Yes	N/A	Yes
Neu et al. 2014B	Yes	No	Yes	Yes	Yes	Yes	Yes	Yes	Yes	Yes
Neu et al. 2015	U	No	Yes	N/A	N/A	Yes	Yes	Yes	N/A	Yes
Reeves et al. 2006	Yes	No	Yes	N/A	N/A	Yes	Yes	Yes	N/A	No
Sharpley et al. 1997	Yes	No	Yes	N/A	N/A	Yes	Yes	Yes	N/A	No
Togo et al. 2008.	Yes	No	Yes	N/A	N/A	Yes	Yes	Yes	N/A	Yes
Togo et al. 2013.	Yes	No	Yes	N/A	N/A	Yes	No	Yes	N/A	Yes
Watson et al. 2003	Yes	Yes	Yes	N/A	N/A	Yes	Yes	Yes	N/A	No
Watson et al. 2004	Yes	Yes	Yes	N/A	N/A	Yes	Yes	Yes	N/A	No

Items answered as not applicable were removed from the final percentage. Abbreviations: JBI, Joanna Briggs Institute; Y, Yes; N, No; N/A, not applicable; U, unclear.

JBI Checklist items:

1. Were the groups comparable other than the presence of disease in cases or the absence of disease in controls?
2. Were cases and controls matched appropriately?
3. Were the same criteria used for identification of cases and controls?
4. Was exposure measured in a standard, valid and reliable way?
5. Was exposure measured in the same way for cases and controls?
6. Were confounding factors identified?

7. Were strategies to deal with confounding factors stated?
8. Were outcomes assessed in a standard, valid and reliable way for cases and controls?
9. Was the exposure period of interest long enough to be meaningful?
10. Was appropriate statistical analysis used?

Armitage et al. 2009

1. Healthy controls and participants are monozygotic twins therefore are age and sex-matched.
2. Recruited via Chronic Fatigue twin registry. Twins were required to be reared together and travel together to Seattle for the study.
3. All patients met the CDC Fukuda definition of ME/CFS. All controls didn't.
4. No exposure.
5. As above.
6. Yes, confounding variables include intermitting sound and temperature. Adjustment of time zones.
7. These were controlled through use of temperature controlled, sound attenuated rooms. Participants were given a week to adjust to their set sleep schedule especially if travel to Seattle was required.
8. Measured using EEG
9. No exposure.
10. Statistical tests include MANOVA, T-tests and Chi square tests. Did not consider multiple comparisons.

Ball et al

1. Patients and controls were monozygotic twins, age- and sex- matched.
2. Twins were reared together and were required to travel to the site together
3. ME/CFS twin met the Center for Disease Control and Prevention ME/CFS criteria (had symptoms of fatigue for at least 6 months) and healthy twin did not have any symptoms of ME/CFS.
4. No exposure, study assessed objective measures of sleep.
5. As above.
6. Alcohol, caffeine, and all medications known to effect fatigue
7. Alcohol, caffeine and medications were discontinued 2 weeks prior to evaluation.
8. Yes, PSG was used.
9. No exposure.
10. *T* tests or McNemar's test was used to assess significant differences between ME/CFS patients and healthy twin. No details on whether normalisation of datasets was considered or whether multiple analyses were accounted for.

Bileviciute-Ljungar et al

1. ME/CFS patients were age and sex- matched.
2. Location data not provided.

3. ME/CFS patients met Fukuda or CCC criteria. HC did not have ME/CFS or any psychiatric disorders, chronic pain, allergies/asthma, sleep disorders or be on sick leave. They also had a high score in health- related quality of life.
4. No exposure, study assessed emotional awareness and sleep quality in ME/CFS patients compared to HC.
5. As above.
6. Did not control for confounding factors.
7. As above.
8. Yes, PSG was used.
9. No exposure.
10. Nonparametric tests (Mann-Whitney U) were used for group comparisons of ordinal data from questionnaires, and two-tailed t-tests were used for age. Multiple analysis was catered for using a stricter criterion for significance. a p-value less than 0.01 was considered a significant correlation

Decker et al. 2009

1. Subjects were Age, sex, BMI and race matched.
2. Participants were race matched and lived in Wichita in 1997
3. All patients conformed to US Center for Disease Control and Prevention criteria for CFS (Fukuda et al., 1994). Participated in the surveillance program. Did not exhibit any medical or psychiatric exclusion and had never reported fatigue of at least 1-month duration.
4. No exposure. Study investigated electroencephalographic correlates of ME/CFS compared to HCs using polysomnography.
5. As above.
6. Confounding factors identified include: age, sex, race, and body mass index in addition to significant confounding medical or psychiatric conditions.
7. These confounding factors were addressed through matching patients with two HCs that match in age, sex, race and BMI. Participants were also excluded based on significant confounding medical or psychiatric assessment.
8. Yes, polysomnography was used to measure outcomes which is used for many clinical symnographic observations.
9. No exposure.
10. Independent samples t-tests were used to assess values of alpha, delta, theta, beta and sigma between control subjects and ME/CFS subjects. This was measured at two-tailed significance of 0.05.

Gotts *et al*

1. Patients were sex- matched and BMI matched but not age matched.
2. Source population information provided – Bank of participants in Newcastle upon Tyne. ME/CFS patients and HCs matched.
3. ME/CFS patients fulfilled CDC criteria for ME/CFS. HCs were free from any sleep-altering medication. Both groups were excluded if they were seeing a sleep medicine specialist, had a diagnosis of obstructive sleep apnoea, periodic limb movement disorder or narcolepsy, had travelled beyond one time zone within three months of study and shift workers.
4. No exposure, study assessed polysomnography results in ME/CFS patients and HCs.

5. As above.
6. Travelling beyond one time zone within three months of study and shift workers. Sleep altering medications were identified in patients. First night effect.
7. Exclusion of participants that travelled beyond one time zone within three-month period and shift workers. Sleep altering medications were identified in patients, however, not controlled for. First night- effect was addressed through completing three nights of polysomnographic recordings.
8. Yes, PSG was used.
9. No exposure.
10. Statistical analysis comprised of independent sample t-test to examine significant differences in demographic and PSG- derived variables. Multiple regression analysis was used to assess the extent that sleep predicted patient daytime symptoms.

Kishi et al 2008

1. Patients were sex matched. Unclear if age matched.
2. Source information not provided.
3. Patients fulfilled the 1994 case definition of ME/CFS this includes: that symptoms can't be explained by physical examination or psychiatric diagnosis. HCs reported health as excellent or good and exam and blood test results were normal.
4. No exposure. Study assessed sleep stage transitions in ME/CFS patients and HCs.
5. As above.
6. Menstruation, strenuous exercise, alcohol and caffeine, rooms where the experiment are hosted.
7. Menstruating women were assessed during follicular phase to reduce variability, participants refrained from alcohol and caffeine the day of the experiment and rooms with equivalent lighting.
8. Yes, PSG was used.
9. No exposure.
10. MANOVA was used and Bonferroni corrections were performed. Statistical significance was accepted when $P < 0.05$.

Le Bon et al. 2008

1. BMI matched but not age matched. Unclear if sex matched.
2. Source population information not provided.
3. CDC criteria was used to recruit patients. To exclude overlap with a primary sleep or psychiatric disorder, further exclusion include: Apnea- Hypopnea index (AHI) or periodic limb movement (PLM) index. Inclusion criteria for HCs were regular sleep schedules, absence of sleep related complaints or regular naps. Additional exclusion criteria include: psychiatric illness as measured by DSM-IV axis I disorder; significant somatic conditions; complaints of excessive daytime sleepiness; presence of an AHI or periodic limb movement.
4. No exposure, study investigated Paradoxical NREMS distribution in ME/CFS patients.
5. As above.
6. Confounding factors include: AHI or PLM index, increased alcoholic intake, consumption of drugs including sleep influencing psychotropic drugs, shift work, daytime napping
7. Patients and HCs that presented with a high AHI or PLM index were excluded. Those who had more than 10 drinks per week or took illicit drugs were also excluded. Daytime napping was not permitted. Those who completed shift work or had a transmeridian flight three weeks prior to the study were excluded.

8. Yes, PSG was used.
9. No exposure.
10. ANOVA with post-hoc tests were used.

Le Bon et al. 2012

1. Exact match on gender and within 2-year age difference between ME/CFS patients and HCs.
2. Location radius not clear. Referred to the sleep unit of the Brugmann University Hospital by the medical check-up. Locally recruited HCs.
3. CDC criteria were used for a first selection of CFS patients. For HCs no significant somatic condition and no current or past mental disorder were allowed. Further exclusion criteria were identical to patient groups.
4. No exposure. Study investigates ultra-slow delta power using EEG in ME/CFS patients and HCs.
5. As above.
6. Confounding factors identified: unauthorised sleep- wake schedules. Alcohol consumption (greater than 2 units per day) Caffeine-including beverages.
7. This was monitored using sleep diaries, but no participants needed to be excluded on this basis. Those who had alcohol consumption greater than 2 units per day were excluded. Caffeine beverages were not allowed post 3pm.
8. Yes, EEG was used to measure delta activity in participants.
9. No exposure.
10. Independent samples t-tests were used to assess values of Ultra-Slow, Delta, Theta, Alpha, Sigma and Beta between CFS patients and HCs. Effect size was measured using Cohen's D. Group differences were considered significant at two-tailed significance of 0.05.

Majer et al. 2007

1. ME/CFS patients and HCs were matched on sex, age, race/ethnicity, and body mass index.
2. Location radius: Wichita, Kansas
3. ME/CFS patients were defined according to the 1994 CDC Fukuda definition. HCs did not have a medical or psychiatric exclusion and did not report fatigue of at least 1-month duration.
4. No exposure. Study investigated objective sleep characteristics in ME/CFS patients using polysomnography and MSLT.
5. As above.
6. Confounding factors: exclusionary medical conditions for ME/CFS e.g. narcolepsy, medications, psychiatric conditions.
7. Exclusion of medical conditions that can explain symptoms, restriction of medication classes that affect sleep and exclusion of participants with severe psychiatric disorders including substance abuse.
8. Yes, PSG and MSLT were used.
9. No exposure.
10. ANOVA and paired sample *t- tests* were used. To account for the risk of type one error scores were adjusted using Bonferroni correction, least squares method and by computing a false discovery rate.

Neu et al. 2007

1. Age, BMI and gender matched
2. Location radius only provided for HCs – locally recruited.
3. ME/CFS patients were defined according to the Fukuda criteria. Further exclusion including for PSD or psychiatric disorders were applied. HCs needed to have regular sleep and wake schedules. No shift work was allowed. No significant somatic condition or present or past mental disorders.
4. No exposure. Study investigated the relationship between sleep efficiency, affective symptoms and intensity of fatigue.
5. As above.
6. Confounding factors include: Confounding factors include: neuropharmacological treatment, night shift workers.
7. Confounding factors were addressed through exclusion.
8. Yes, PSG was used.
9. No exposure.
10. Used parametric tests for all but PSQI subscales, where datasets weren't parametric a mann whitney U test was used. Between group comparisons assessed using MANOVA with Bonferroni correction.

Neu et al. 2008

1. Age, BMI and sex- matched.
2. Location radius only provided for HCs – locally recruited.
3. ME/CFS patients were defined according to the Fukuda criteria. Further exclusion including for PSD or psychiatric disorders were applied. HCs needed to have regular sleep and wake schedules. No shift work was allowed. No significant somatic condition or present or past mental disorders.
4. No exposure. Study investigated the relationship between sleepiness and fatigue.
5. As above.
6. Confounding factors include: neuropharmacological treatment, night shift workers. Age and BMI.
7. Confounding factors were addressed through exclusion. Age and BMI were expressed as covariates.
8. Yes, PSG and MSLT
9. No exposure.
10. MANCOVA with single factor and two covariates were used. Tested at 5% significance level. While there were considerations made for parametric tests, no considerations for multiple comparisons were made.

Neu et al. 2014A

1. ME/CFS patients and controls show no difference in age, sex or BMI.

2. No location radius was provided.
3. ME/CFS participants were defined according to CDC Fukuda criteria. No clear criteria were provided for HCs.
4. No exposure. Study investigates non-REM sleep EEG power distribution in CFS/ME patients compared to HCs.
5. As above.
6. Confounding factors include: hypnotics or other relevant neuropsychopharmacological (including antidepressants) and sleeping time
7. Cease neuropsychopharmacological (including antidepressants) for at least 2 weeks prior to recording. Participants required a minimum time in bed (TIB) threshold of 300min and a minimum total sleep time (TST) threshold of 180min.
8. Yes, EEG was used.
9. No exposure.
10. Hypothesis tests were performed two-sided at the 5% significance level. A Kolmogorov- Smirnov test was used to assess normality for continuous variables. Non-normal distributed data was rank converted. Between group comparisons were assessed using MANCOVA. Posteriori tests were performed using a sequential Bonferroni (Dunn–Sidak) correction for multiple comparisons.

Neu et al. 2014B

1. ME/CFS patients and controls show no difference in age, sex or BMI.
2. No location radius was provided.
3. ME/CFS participants were defined according to CDC Fukuda criteria. Participants were not included if there could be a factor contributing to fatigue or sleep e.g. oncological conditions, autoimmune affection, infectious disease etc. High BMI (> 28), high alcohol or coffee consumption in patients or control groups.
4. Exposure was a muscular hand grip task and cognitive, vigilance and psychomotor assessments. Visual stimuli that appears at random intervals – participants respond by pressing a space bar. Finger tapping task, symbol span or digit symbol substitution test and Zazzo's cancellation task.
5. All tests are validated and were consistent in their approach to measuring cognitive, vigilance and psychomotor responses and muscular strength.
6. Confounding factors include: hypnotics or other relevant neuropsychopharmacological (including antidepressants) and sleeping time. Alcohol (greater than 2 units per day).
7. Cease neuropsychopharmacological (including antidepressants) for at least 2 weeks prior to recording. Exclusion of participants with alcohol intake that is greater than two units per day.
8. Yes, PSG and MSLT were used.
9. This study uses standardised tests and time points that have been previously validated.
10. Normality was assessed using Kolmogorov–Smirnov tests were selected on the basis of this result. Between group comparisons using MANOVA with a single factor (subject group) was computed. When there was a violation of sphericity, Greenhouse–Geisser

corrections were used. Stepwise forward linear multivariate regression analysis was used to assess predictors of fatigue severity.

Neu 2015

1. Age and BMI matched unclear if sex matched.
2. Source population not provided.
3. ME/CFS patients were diagnosed according to the CDC Fukuda criteria. Subjects were excluded if they had any confounding illness that may explain their fatigue e.g. primary sleep disorder. HCs had no significant somatic conditions including current or past mental disorders.
4. No exposure. Study investigated slow wave sleep and power spectra distribution patterns in ME/CFS patients compared to HCs.
5. No exposure.
6. Confounding factors include: hypnotics or other relevant neuropsychopharmacological (including antidepressants) and sleeping time. Alcohol (greater than 2 units per day).
7. Cease neuropsychopharmacological (including antidepressants) for at least 2 weeks prior to recording. Exclusion of participants with alcohol intake that is greater than two units per day.
8. Yes, PSG was used
9. No exposure.
10. MANOVA was used for normally distributed demographic, psychometric and polysomnographic variables while for non-normally distributed variables Kruskall-Wallis H and Mann-Whitney U tests were used. Mixed ANOVA was used to assess relative power spectrum distributions. Violation of sphericity was assessed using Mauchly's test. Greenhouse- geisser tests were reported where the violation occurs. Bonferroni post hoc tests were conducted.

Reeves et al

1. ME/CFS patients and HCs were matched for sex, race, age and body mass index.
2. Participants were race matched, however, demographic matching was not maintained throughout the duration of the study.
3. ME/CFS patients were recruited according to the Fukuda criteria. HCs did not have medical/ psychiatric exclusions and have not had a previous history of fatigue for at least one month.
4. No exposure. Study investigated sleep characteristics of people with ME/CFS compared to HCs.
5. As above.
6. Confounding factors include: exclusionary sleep disorders for ME/CFS, substance abuse, use of sleep impacting medications.
7. Those with sleep disorders that may describe symptoms were excluded. Those with substance abuse within the last 5 years prior to recruitment were also excluded. Use of sleep impacting medications were accounted for at the level of analysis.
8. Yes, PSG and MSLT was used.

9. No exposure.
10. Standard and exact logistic regression models were used to compute odds ratios of estimates of relative requests and 95% confidence intervals. Chi square statistic was used to assess associations between ME/CFS and dichotomous variables. No considerations were made for selection of tests based on normality of datasets nor was adjustments for multiple comparisons made.

Sharpley et al

1. ME/CFS patients were matched for age and sex.
2. Source population information not provided.
3. ME/CFS patients were recruited according to the Fukuda criteria and ICD-10 criteria for pure ME/CFS. HCs had no significant current or lifetime psychiatric or medical illness. Those who took psychotropic medication were also excluded.
4. No exposure. Study investigated abnormal sleep in ME/CFS patients compared to HCs.
5. As above.
6. Confounding factors: obstructive sleep apnoea and narcolepsy, those who met criteria for DSM-III-R diagnoses of major depression, dysthymia, panic disorder, agoraphobia, generalized anxiety disorder or those who take psychotropic medication.
7. Participants with a primary sleep disorder eg: obstructive sleep apnea and narcolepsy were excluded. Those who had a psychiatric illness or took psychotropic medication were also excluded.
8. Yes, PSG was used.
9. No exposure.
10. Sleep complaints were presented as percentages. Objective sleep parameters were presented as means. ME/CFS and HCs were then compared using unpaired T tests. No adjustments for multiple comparisons were made nor was reasons for selection of tests detailed.

Togo et al (2008)

1. Age, sex and body mass index matched.
2. Source population information not provided.
3. ME/CFS patients were recruited according to Fukuda criteria. Patients had no alternative explanation from their symptoms such as history, physical examination, or laboratory tests. Psychiatric interview confirmed that participants did not have the following conditions: schizophrenia, eating disorders, substance abuse, bipolar, major depressive disorder, or a psychiatric disorder that may disrupt sleep. HCs also followed these exclusionary criteria. No other details as to what defines a HC was described.
4. No exposure. Study investigated sleep structure and sleepiness in ME/CFS patients with or without coexisting fibromyalgia compared to HCs.
5. As above.
6. Confounding factors include: alcohol, Caffeine, exercise, sleep architecture, strenuous exercise. Rooms.

7. All participants refrained from alcohol and caffeine ingestion and strenuous and prolonged exercise in the morning of study nights. Rooms were consistently quiet and shaded.
8. Yes, PSG was used.
9. No exposure.
10. Differences in measured variables were assessed using a student T test or ANOVA. Post hoc analyses including Turkey student range test were conducted to adjust for multiple comparisons.

Togo et al (2013)

11. Age, sex and body mass index matched.
12. Source population information not provided.
13. ME/CFS patients were recruited according to Fukuda criteria. Patients had no alternative explanation from their symptoms such as history, physical examination or laboratory tests. Psychiatric interview confirmed that participants did not have the following conditions: schizophrenia, eating disorders, substance abuse, bipolar, major depressive disorder, or a psychiatric disorder that may disrupt sleep. HCs also followed these exclusionary criteria. No other details as to what defines a HC was described.
14. No exposure. Study investigated heart rate variability during sleep and sleepiness in ME/CFS patients compared to HCs.
15. As above.
16. Confounding factors include: alcohol, Caffeine, exercise, sleep architecture, menstrual times. Rooms.
17. All participants refrained from alcohol and caffeine ingestion and strenuous and prolonged exercise in the morning of study nights. Participants all were studied during their follicular stage in the menstrual cycle. Rooms were consistently quiet and shaded. Effects of sleep architecture were not controlled for in this study.
18. Yes, PSG was used.
19. No exposure.
20. Differences in measured variables were assessed using a student T test or ANOVA. Post hoc analyses were conducted to adjust for multiple comparisons.

Watson et al (2003)

1. ME/CFS patients and HCs were monozygotic twins – sex and age matched.
2. Participants were reared together and travelled to site together.
3. ME/CFS patients were recruited according to the Fukuda criteria. Twin that did not have ME/CFS were healthy and had same psychiatric and medical inclusion/ exclusion criteria as twin with ME/CFS.
4. No exposure. Study investigated subjective and objective sleepiness in monozygotic twins discordant for ME/CFS.
5. As above.
6. Confounding factors include: HIV, alcohol, caffeine and medications.

7. Participants with HIV were excluded. Participants were requested to abstain from alcohol and caffeine and all medications were discontinued two weeks prior to the evaluation.
8. Yes, PSG was used.
9. No exposure.
10. No description of appropriateness of selected tests. Adjustments for multiple comparisons were not made.

Watson et al (2004)

1. ME/CFS patients and HCs were monozygotic twins – sex and age matched.
2. Participants were reared together and travelled to site together.
3. ME/CFS patients were recruited according to the Fukuda criteria. Twin that did not have ME/CFS were healthy and had same psychiatric and medical inclusion/ exclusion criteria as twin with ME/CFS.
4. No exposure. Study investigated subjective and objective sleepiness in monozygotic twins discordant for ME/CFS.
5. As above.
6. Confounding factors include: HIV, alcohol, caffeine and medications.
7. Participants with HIV were excluded. Participants were requested to abstain from alcohol and caffeine and all medications were discontinued two weeks prior to the evaluation.
8. Yes, PSG and MSLT
9. No exposure.
10. No description of appropriateness of selected tests. Adjustment for multiple comparisons were not made.
