# Supplementary material for: Systematic Review of Sleep Characteristics in Myalgic Encephalomyelitis/Chronic Fatigue Syndrome
Source: Healthcare (Basel). 2021 May 11;9(5):568. doi: 10.3390/healthcare9050568 (PMC8150292; doi:10.3390/healthcare9050568)
Supplement: Supplementary file 1 [file healthcare-09-00568-s001.zip › Supplementary Files/Table S1. Search code.pdf]

Table S1. Search code

Database	Code
Pubmed	(("Fatigue Syndrome, Chronic"[Mesh])) AND (("Polysomnography"[Mesh]) OR ("Polysomnograph*") OR ("Multiple Sleep Latency Test"))
Medline, Embase and Web of Science	(("Chronic Fatigue Syndrome") OR ("Myalgic Encephalomyelitis") OR (“Encephalomyelitis, Myalgic”) OR (“Chronic Fatigue Syndromes”) OR (“Fatigue Syndromes, Chronic”) OR (“Chronic Fatigue-Fibromyalgia Syndrome”) OR (“Chronic Fatigue Fibromyalgia Syndrome”) OR (“Chronic Fatigue Fibromyalgia Syndromes”) OR (“Fatigue-Fibromyalgia Syndrome, Chronic”) OR (“Fatigue-Fibromyalgia Syndromes, Chronic”) OR (“Postviral Fatigue Syndrome”) OR (“Infectious Mononucleosis-Like Syndrome, Chronic”) OR (“Infectious Mononucleosis Like Syndrome, Chronic”) OR (“Royal Free Disease”) OR (“Chronic Fatigue and Immune Dysfunction Syndrome”) OR (“Chronic Fatigue Disorder”) OR (“Chronic Fatigue Disorders”) OR (“Fatigue Disorder, Chronic”) OR (“Fatigue Disorders, Chronic”) OR ("systemic exertion intolerance") OR (“Fatigue Syndrome, Postviral”) OR (“Fatigue Syndromes, Postviral”) OR (“Postviral Fatigue Syndromes”)) AND ((“Polysomnography”) OR (“Polysomnographies”) OR (“Monitoring, Sleep”) OR (“Sleep Monitoring”) OR (“Somnography”) OR (“Somnographies”) OR (“Polysomnograph*”) OR (“Multiple Sleep Latency Test”))
