# Supplementary material for: Systematic Review of Sleep Characteristics in Myalgic Encephalomyelitis/Chronic Fatigue Syndrome
Source: Healthcare (Basel). 2021 May 11;9(5):568. doi: 10.3390/healthcare9050568 (PMC8150292; doi:10.3390/healthcare9050568)
Supplement: Supplementary file 1 [file healthcare-09-00568-s001.zip › Supplementary Files/Table S2. Study and Patient Characteristics.pdf]

Table S2. Study and Patient Characteristics

**Author & year**	**Dx**	**Study design**	**Subjects**		**Age (years, mean ± SD)**		**Sex, female (%)**		**BMI (kg m−2, mean ± SD)**		**Total sleep duration (min ± SD)**		**Illness duration (years)**	**Method of analysis**
			ME/CFS	HC	ME/CFS	HC	ME/CFS	HC	ME/CFS	HC	ME/CFS	HC		
Armitage et al 2009	Fukuda (1994)	Prospective Observational twin study	13	13	45.4 ± 10.0	NR	100%	NR	30.1	29.7	395.9 ± 53.4	403.9 ±35.8	9.2	PSG
Ball et al 2004	Fukuda (1994)	Prospective Observational twin study	22	22	41.4	NR	90.9%	NR	NR	NR	376.2 ± 43.1	383.1 ± 61.1	7.0	PSG
Bileviciute-Ljungar et al 2020	Fukuda (1994) & CCC (2003)	Prospective Observational case-control	23	30	43 ± 12.0	45 ± 12.0	78.3%	70%	24 ± 3.0	NR	NR	NR	NR	PSG
Decker et al 2009	Fukuda (1994)	Prospective Observational case-control	35	40	male 47.0 ± 0.7, female 50.9 ± 9.1	Male 48.0 ± 10.9, female 50.8 ± 8.1	85.7%	90%	male 28.2 ± 5.4, female 28.8 ± 4.1	Male: 29.5 ± 5.7, female: 29.4 ± 8.1	NR	NR	>5	PSG MSLT
Gotts et al 2016	Fukuda (1994)	Prospective Observational case-control	19	22	44.63 ± 9.74	25.18 ± 5.86	73.7%	50%	26.09 ± 3.89	25.18 ± 3.57	442.39 ± 85.85	430.43 ± 30.08	NR	PSG
Kishi et al 2008	Fukuda (1994)	Prospective Observational case-control	22	22	42 ± 8.0	38 ± 8	100%	100%	NR	NR	NR	NR	NR	PSG
Le Bon et al 2008	Fukuda (1994)	Prospective Observational case-control	28	27	33.2 ± 8.7	37.4 ± 5.6	89.3%	37%	23.2 ± 3.2	23.1 ± 4.1	364.9 ± 60.4	400.5 ±37.1	NR	PSG
Le Bon et al 2012	Fukuda (1994)	Prospective Observational case-control	10	10	30.7 ± 8.0	32.2 ± 9.8	100%	100%	22.9 ± 4.3	21.5 ± 1.9	455.6 ± 54.7	458.5 ± 47.2	NR	PSG

Majer et al 2007	Fukuda (1994)	Prospective Observational case-control	43	43	50.3	50.5	85.7%	90%	28.7	29.2	400.3	407.9	NR	PSG MSLT
Neu et al 2007	Fukuda (1994)	Prospective Observational case-control	28	12	34.2 ± 9.2	32.3 ± 9.6	100%	100%	23.1 ± 4.1	21.3 ± 2.0	433.18 ± 71.8	406.18 ± 32.5	NR	PSG
Neu et al 2008	Fukuda (1994)	Prospective Observational case-control	16	12	32.8	32.2	100%	100%	23.1 ± 4.3	21.3 ± 1.9	NR	NR	NR	PSG MSLT
Neu et al 2014A	Fukuda (1994)	Prospective Observational case-control	52	25	44.8 ± 1.9	40.46 ± 2.4	88.5%	72%	24.5 ± 0.9	24.71 ± 1.1	358.00 ± 11.3	386.62 ± 13.8	NR	PSG
Neu et al 2014B	Fukuda (1994)	Prospective Observational case-control	15	14	35.13 ± 4.8	31.50 ± 8.1	81.25%	71.4%	24.09 ± 3.4	22.03 ± 1.9	416.36 ± 88.1	398.96 ± 57.7	NR	PSG MSLT
Neu et al 2015	Fukuda (1994)	Prospective Observational case-control	30	22	44.36 ± 9.7	38.45 ± 14.2	86.7%	72.7%	24.55 ± 4.9	23.90 ± 4.8	387.32 ± 40.7	389.23 ±49.8	NR	PSG
Reeves et al 2006	Fukuda (1994)	Prospective Observational case-control	43	43	50.6	50.3	83.7%	88.4%	29.4	29.3	400.3	407.9	7.3	PSG MSLT
Sharpley 1997	Fukuda (1994)	Prospective Observational case- control	20	20	NR	NR	50%	50%	NR	NR	410 ± 14.2	409 ± 12.9	1.5	PSG
Togo *et al* 2008	Fukuda (1994)	Prospective Observational case- control	53	42	39 ± 8	38 ± 8	60.4%	71.4%	24.9 ± 5.2)	24.4 ± 4.8	385 ± 39	351 ±54	NR	PSG
Togo and Natelson 2013	Fukuda (1994)	Prospective Observational case- control	18	19	39 ± 8.0	37 ± 9	100%	100%	22.9 ± 3.3	25.8 ± 6.5	352 ± 55	391 ± 38	NR	PSG
Watson et al 2003	Fukuda (1994)	Prospective Observational twin study	22	22	41.4	NR	90.9%	NR	NR	NR	378	378	7.0	PSG
Watson et al 2004	Fukuda (1994)	Prospective Observational twin study	20	20	41	NR	90%	NR	NR	NR	NR	NR	7.0	PSG MSLT

Abbreviations: BMI, Body Mass Index; CCC, Canadian Consensus Criteria; Dx, Diagnostic Criteria; HC, healthy control; ME/CFS, Myalgic Encephalomyelitis/ Chronic Fatigue Syndrome; MSLT, Multiple Sleep Latency Testing; NR, Not Recorded; PSG, Polysomnography; SD, Standard Deviation
