# Supplementary material for: Systematic Review of Sleep Characteristics in Myalgic Encephalomyelitis/Chronic Fatigue Syndrome
Source: Healthcare (Basel). 2021 May 11;9(5):568. doi: 10.3390/healthcare9050568 (PMC8150292; doi:10.3390/healthcare9050568)
Supplement: Supplementary file 1 [file healthcare-09-00568-s001.zip › Supplementary Files/Table S3. Summary of primary outcome measures.pdf]

Table S3. Summary of primary outcome measures

Author (date)	Technique	Findings
Armitage *et al.* (2009)	PSG	No significant differences in spectral power in any frequency band including REM latency, delta-wave, fast frequency beta or alpha power were found in the twin with ME/CFS compared to the healthy twin. There, however, were significant differences in sleep stage main effect (p<0.001), EEG frequency band main effect (p < 0.001) and sleep stage by frequency band interaction (p< 0.0001) in ME/CFS patients compared to the healthy twin.
Ball *et al.* (2004)	PSG	Significantly increased stage 3 (p≤0.05) and REM sleep were found in ME/CFS patients compared to their healthy twin. ME/CFS patients also showed higher AHI (p≤0.05) and apnoea hypopnea arousal index (p≤0.05) values compared to the healthy twin. No significant differences were observed in sleep architecture including sleep latency, REM latency and total sleep time.
Bileviciute-Ljungar et al (2020).	PSG	All ME/CFS patients had reduced total sleep time. Higher sleep latency and number of awakenings was found in most patients (78% and 70% respectively). In ME/CFS patients, 61% of N1 stage was higher and 57% of REM was lower. Arousal index was impaired in 22% of patients.
Decker *et al.* (2009)	PSG<br>MSLT	Alpha power in the ME/CFS cohort was diminished during stage 2, slow wave, and REM sleep (p < 0.0001) compared to HC. Delta power decreased during slow wave sleep (p < 0.0001) but was elevated during stage 1 (p < 0.0001) and REM (p < 0.001). Theta, sigma and beta spectral power during stage 2, slow wave sleep and REM were significantly reduced in patients compared to HCs (p<0.0001).
Gotts *et al. (*2016)	PSG	ME/CFS patients showed significantly increased wake after sleep onset (p<0.05), percentage wake (p<0.01) and REM latency (p<0.05) compared to HCs. ME/CFS patients showed more severe symptoms of insomnia compared to HCs (p<0.001). ME/CFS patients showed decreased percentage sleep efficiency index compared to HCs (p<0.05).
Kishi *et al.* (2008)	PSG	REM to non-REM sleep transitions have been detected in humans. The frequency of these transitions was found to be significantly reduced in ME/CFS patients compared to HC (p<0.01). Subsequent alteration of transition patterns also occurs resulting in significantly greater relative transition frequency of moving from both REM and stage I sleep to awake (p<0.01).
Le bon *et al.* (2007)	PSG	ME/CFS patients had significantly lower sleep period time (p<0.017), total sleep time (p<0.009), sleep efficiency index (p<0.013), movement time (p<0.001) and REM sleep latency (p<0.055) and higher sleep onset latency (p<0.0001) compared to ME/CFS patients.
Le Bon *et al.* (2012)	PSG	The ultra-slow delta power was approximately one fifth lower in ME/CFS patients compared to HCs in N3 sleep (p=0.046). Theta, alpha, sigma and beta did not differ significantly between the two groups. ME/CFS patients had significantly higher MAI (p=0.031) and N3 sleep % (p=0.026) compared to HCs.
Majer (2007)	PSG<br>MSLT	There were no significant differences found in sleep pathology or architecture parameters measured using MSLT and PSG in ME/CFS patients compared to HCs.

Neu (2007)	PSG	Only two objective sleep parameters the MAI (p<0.001) and AHI per hour (p<0.095) were significantly higher in ME/CFS patients compared to HCs.
Neu (2008)	PSG<br>MSLT	ME/CFS patients showed significantly increased sleep onset latency (p=0.042), AHI per hour of sleep (p<0.0001) and micro arousal index hour of sleep (p<0.0001) compared to HCs. ME/CFS patients showed significantly decreased scores on the MSLT (p<0.005) compared to HCs.
Neu (2014A)	PSG	ME/CFS patients showed significantly increased WASO (p=0.033), SWS (p=0.015), MAI (p<0.0001), AHI (p <0.001) and periodic limb movement (p=0.035) compared to HCs.
Neu (2014B)	PSG<br>MSLT	ME/CFS patients had significantly longer SWS duration as well as higher periodic limb movements and AHI (p < 0.001). No significant difference between patient and HC groups were found in MSLT sleep latencies, sleep period time, total sleep time, non-REM stages N1 and N2, REM latency and duration, and sleep efficiency.
Neu (2015)	PSG	ME/CFS patients had significantly greater SWS (p=0.024) and MAI (p<0.001) scores compared to HC. There was a significant decrease in central ultra-slow power (0.3-0.79Hz) in the patient group (p<0.001).
Reeves (2006)	PSG<br>MSLT	There were no differences of clinical significance in frequency of obstructive sleep apnoea per hour in ME/CFS patients. There were also no significant differences in sleep architecture characteristics in ME/CFS patients compared to HCs.
Sharpley (1997)	PSG	There were no significant differences found in sleep characteristics between ME/CFS patients and HC. There was, however, a significant decrease in sleep efficiency (p<0.05) and increase in time spent in bed (p<0.05) and total wake time after start of sleep (p<0.05) in ME/CFS patients compared to HC.
Togo (2008)	PSG	ME/CFS patients had significantly different PSG recordings and subjective experiences compared to HC (p < 0.05). Differences also include less total sleep time (p < 0.05), lower sleep efficiency (p < 0.05) and less rapid eye movement in the ME/CFS patient group (p < 0.05). ME/CFS patients were stratified into a.m. sleepier and a.m. less sleepy groups. ME/CFS patients that were in the a.m. sleepier group had reduced length of uninterrupted sleep (p < 0.05).
Togo (2013)	PSG	Although PSG results are not significantly different between ME/CFS patients and HC, patients reporting more sleepiness in the morning showed significantly higher (p<0.05) fractal scaling index alpha 1 during stages 1, 2 and 3 sleep. In patients, there was a correlation between self-reported sleepiness and fractal scaling index alpha 1 during non-REM sleep (~r=0.51, p<0.05) Fractal scaling index alpha 1 was significantly lower when asleep for HC and patients in the less sleepy group (p< 0.05), however, these values did not change in the sleepier group.
Watson (2003)	PSG	ME/CFS patients presented with eight subjective measures of insomnia and low-quality sleep (p≤0.05). Percent stage REM sleep was higher in the ME/CFS twin compared to the healthy twin (p≤0.05). Other objective measures including sleep latency, total sleep time, sleep efficiency, arousal number, arousal index, hypnogram awakenings, REM- sleep latency did not significantly differ between the two groups.
Watson (2004)	PSG<br>MSLT	There were no significant differences found between the two groups in mean sleep latency on the MSLT.

Abbreviations: AHI, apnoea- hypopnea index; HC, healthy controls; ME/CFS, Myalgic Encephalomyelitis/ Chronic Fatigue Syndrome; MAI, Micro-arousal index; MSLT, Multiple Sleep Latency Testing; PSG, Polysomnography; REM, Rapid Eye Movement; SWS, Slow Wave Sleep
