# Supplementary material for: Systematic Review of Sleep Characteristics in Myalgic Encephalomyelitis/Chronic Fatigue Syndrome
Source: Healthcare (Basel). 2021 May 11;9(5):568. doi: 10.3390/healthcare9050568 (PMC8150292; doi:10.3390/healthcare9050568)
Supplement: Supplementary file 1 [file healthcare-09-00568-s001.zip › Supplementary Files/Table S4. Summary of secondary outcome measures.pdf]

Table S4. Summary of secondary outcome measures

	Secondary outcome measure (s)	Results
Armitage et al (2009)	N/A	N/A
Ball et al (2004)	N/A	N/A
Bileviciute et al (2020)	TAS-20 DIF<br>TAS-20 DDF<br>TAS-20 EOT<br>TAS-20 EOT<br>TAS Total<br>LEAS-Self<br>LEAS-Other<br>LEAS-Total<br>HADS-D<br>HADS-A	TAS-20 DIF: p <0.001<br>TAS-20 DDF: NS<br>TAS-20 EOT: NS<br>TAS-20 EOT: NS<br>TAS Total: p <0.05<br>LEAS-Self: p <0.01<br>LEAS-Other: NS<br>LEAS-Total: NS<br>HADS-D: p <0.001<br>HADS-A: NS
Decker et al (2009)	N/A	N/A
Fischler et al (1997)	N/A	N/A
Gotts et al (2016)	PSQI subjective sleep quality<br>PSQI sleep latency<br>PSQI sleep duration<br>PSQI habitual sleep efficiency<br>PSQI sleep disturbances<br>PSQI use of sleep medication<br>PSQI daytime dysfunction<br>PSQI global<br>ISI	PSQI subjective sleep quality: p <0.001<br>PSQI sleep latency: p <0.001<br>PSQI sleep duration: p <0.05<br>PSQI habitual sleep efficiency: p <0.05<br>PSQI sleep disturbances: p <0.001<br>PSQI use of sleep medication: NS<br>PSQI daytime dysfunction: p < 0.01<br>PSQI global: p <0.001<br>ISI: p <0.001
Kishi et al (2008)	N/A	N/A
Le Bon et al (2007)	N/A	N/A
Le Bon et al 2012)	PSQI<br>FSS<br>ESS	PSQI: p <0.001<br>FSS: p <0.001<br>ESS: 0.010
Majer et al (2007)	Insomnia<br>Sleepiness<br>Physical/ somatic<br>Apnea<br>Body clock<br>Nasal obstruction	Insomnia: p <0.001<br>Sleepiness: p = 0.060<br>Physical/ somatic: p = 0.001<br>Apnea: p = 0.865<br>Body clock: 0.610<br>Nasal obstruction: 0.318
Neu et al (2007)	PSQI global score<br>Sleep quality<br>Sleep latency<br>Sleep duration<br>Sleep efficiency<br>Sleep disturbance<br>Use of medication<br>Daytime dysfunction<br>FSS mean score<br>HAMA<br>HAMD_21<br>BDI-13	PSQI global score: p < 0.01<br>Sleep quality: p < 0.01<br>Sleep duration: NS<br>Sleep efficiency: p = 0.017<br>Sleep disturbance: p= 0.004<br>Use of medication: NS<br>Daytime dysfunction: p < 0.01<br>FSS mean score: p < 0.01<br>HAMA: p < 0.01<br>HAMD_21: p < 0.01<br>BDI-13: p < 0.01
Neu et al (2008)	ESS<br>FSS	ESS: p <001<br>FSS: p <001

	BDI<br>HAMA	BDI: p <001<br>HAMA: p <001
Neu et al (2014A)	PSQI<br>ESS<br>FSS<br>HAD-A<br>HAD-D	PSQI: p < 0.001<br>ESS: p < 0.001<br>FSS: p < 0.001<br>HAD-A: p < 0.001<br>HAD-D: p < 0.001
Neu et al (2014B)	HAD-A<br>HAD-D<br>VAS-F<br>VAS-S<br>PSQI<br>FSS<br>ESS	HAD-A: p = 0.009<br>HAD-D: p = 0.001<br>VAS-F: p < 0.001<br>VAS-S: NS<br>PSQI: p < 0.001<br>FSS: p < 0.001<br>ESS: p < 0.006
Neu et al (2015)	HAD-A<br>HAD-D<br>HAD- Total<br>ESS<br>FSS<br>PSQI	HAD-A: p = 0.001<br>HAD-D: p < 0.001<br>HAD- Total: p < 0.001<br>ESS: 0.593<br>FSS: p < 0.001<br>PSQI: p < 0.001
Reeves et al (2006)	N/A	N/A
Sharpley et al (1997)	HAD-A<br>HAD-D<br>BDI-D<br>SF-36<br>FSS	N/A
Togo et al (2008)	CES-D<br>**Likert scale**<br>**Sleepiness:**<br>Evening<br>Morning<br>**Fatigue:**<br>Evening<br>Morning<br>**Pain:**<br>Evening<br>Morning<br>**Feeling blue**<br>Evening<br>Morning	CES-D: p < 0.05<br>**Likert scale**<br>**Sleepiness:**<br>Evening: p < 0.05<br>Morning: p < 0.05<br>**Fatigue:**<br>Evening: p < 0.05<br>Morning: p < 0.05<br>**Pain:**<br>Evening: p < 0.05<br>Morning: p < 0.05<br>**Feeling blue**<br>Evening: NS<br>Morning: p < 0.05
Togo et al (2013)	CES-D<br>**Sleepiness:**<br>Evening<br>Morning	CES-D: p < 0.05<br>**Sleepiness:**<br>Evening: p < 0.05<br>Morning: p < 0.05
Watson et al (2003)	SSS<br>ESS	SSS: p <0.001<br>ESS: p< .05
Watson et al (2004)	I wake up often during the night;<br>My nights sleep is often restless and disturbed;<br>I feel that my sleep is abnormal;<br>I have trouble getting to sleep at night;<br>I have been unable to sleep at all for several days;	I wake up often during the night: p ≤0.001<br>My nights sleep is often restless and disturbed: p ≤0.001<br>I feel that my sleep is abnormal: p ≤0.001<br>I have trouble getting to sleep at night: p ≤0.001<br>I have been unable to sleep at all for several days: p ≤0.05

	I feel that I have insomnia;<br>I often have a poor nights sleep;<br>I take a prescription drug to help me sleep.	I feel that I have insomnia: p ≤0.01<br>I often have a poor nights sleep: p ≤0.001<br>I take a prescription drug to help me sleep: p ≤0.001

Abbreviations: CES-D, Center for Epidemiological Studies- Depression; ESS, Epworth Sleepiness Scale, FSS, HADS, Hospital Anxiety and Depression Scale; HADS-A, Hospital Anxiety and Depression Scale Anxiety; HADS-D, Hospital Anxiety and Depression Scale Depression; HAMA, Hamilton Anxiety Rating Scale; ISI, Insomnia Severity Index; LEAS, Levels of Emotional Awareness Scale; TAS, Toronto Alexithymia Scale; PSQI, Pittsburgh Sleep Quality Index; VAS-F, Visual Analogue Scale- Fatigue; VAS-S, Visual Analogue Scale- sleep;
